# Brain regions associated with frequent occurrences of seizures related to low-grade gliomas: a voxel-level evidence

**DOI:** 10.3389/fnhum.2026.1792225

**Published:** 2026-03-27

**Authors:** Zeya Yan, Renwu Zhang, Tingting Pu, Hongbo Bao, Jie Hu, Ruiyang Wang, Fuqiang Feng, Yinyan Wang

**Affiliations:** 1 Department of Neurosurgery, Beijing Tiantan Hospital, Capital Medical University, Beijing, China; 2 Department of Neurosurgery, Shanxi Province Cancer Hospital, Taiyuan, Shanxi, China; 3 Beijing Neurosurgical Institute, Capital Medical University, Beijing, China

**Keywords:** low-grade glioma, epilepsy, seizure frequency, magnetic resonance imaging, voxel-based mapping

## Abstract

**Background:**

Tumor location has been demonstrated to influence the risk of epilepsy in patients with low-grade gliomas (LGGs). However, existing evidence is mainly based on dichotomized analyses, without adequately accounting for seizure frequency. We present a voxel-wise lesion analysis to investigate the potential correlations between the location of tumor and the frequency of seizure occurrence in patients with LGGs.

**Methods:**

We retrospectively reviewed the clinical information and magnetic resonance images of 352 patients pathologically confirmed with LGGs. Preoperative seizure frequency during the 3 months prior to the primary surgery was classified as daily (≥1/day), weekly (≥1/week), monthly (≥1/month) and rare (<1/month). A voxel-based lesion-symptom mapping analysis was performed to identify the association between tumor location and preoperative seizure frequency.

**Results:**

LGGs involving the left primary motor area or right insular and basal ganglia were associated with an increased risk of frequent seizures (≥1/month). A focal aware seizure was more likely to frequently occur compared with a focal to bilateral tonic–clonic seizure caused by LGGs (*p* < 0.001). Tumors that caused a high seizure frequency were less likely to be completely removed (*p* = 0.008). Furthermore, preoperative seizure frequency was a significant predictor of seizure outcomes at 6 months after surgery as determined by a univariate analysis (*p* = 0.002).

**Conclusion:**

This study utilized voxel-level neuroimaging analyses to identify the anatomical correlations of seizure frequency in patients with LGGs, providing further insight into seizure risk stratification and potential implications for surgical management.

## 1 Introduction

Seizures represent a primary clinical presentation in patients suffering from low-grade gliomas (LGGs) (You et al., 2020; Li et al., 2024; Jiang et al., 2026). A frequent occurrence of seizures can have a severe impact on the patient’s quality of life. An increase in seizure frequency may occur during tumor growth and indicate a loss of control by the anti-seizure medication (ASM), predicting worse seizure outcome after tumor resection (Ryden et al., 2021; Mazzucchi et al., 2022). Many factors have been associated with epileptogenesis in LGGs, including the tumor type, tumor location, peritumoral microenvironment, neural activity, and genetic changes (Slegers and Blumcke, 2020; Grimi et al., 2024; Li et al., 2024; Wang et al., 2025). Nevertheless, the mechanisms that drive a tumor to cause seizures remain unclear.

Clinical studies have suggested an association between the tumor location and the seizure risk, but significant inconsistencies exist among these observations (Pallud et al., 2014; Akeret et al., 2020; Feyissa et al., 2023). Most previous studies calculated the incidence of epilepsy by dichotomizing patients according to whether their tumor involved a single brain lobe; this common approach is limited to the use of dichotomized datasets (Audrey et al., 2024). In our previous study, quantitative analysis was performed to correlate the tumor location and the type of seizure presented in patients (Wang et al., 2015). Here, we made a further effort with exploring the anatomical correlations of the frequency of occurrence of tumor-related seizures using voxel-based analysis.

To address this issue, we performed a voxel-based lesion-symptom mapping analysis (VLSM), which was applied to investigate the anatomical correlation between brain lesions and clinical symptoms (Bates et al., 2003; Hardstone et al., 2025). This approach considers the heterogeneity that may exist in a brain lobe in causing a seizure. In the present study, a voxel-wise regression analysis was performed to localize tumor locations that correlated with the frequency of seizure occurrence in the LGG patients. Our results demonstrate that the LGGs involving the specific brain regions caused an increased frequency of seizure occurrence.

## 2 Materials and methods

### 2.1 Patients characteristics

This retrospective study analyzed a consecutive dataset from the Chinese Glioma Genome Atlas (CGGA) database,^1^ comprising clinical information and magnetic resonance (MR) images. To ensure the robustness of the VLSM analysis and the accuracy of clinical correlations, strict inclusion and exclusion criteria were applied, resulting in a final cohort of 352 LGG patients. Inclusion criteria were: (1) histopathologically confirmed WHO Grade 2 LGG; (2) available preoperative T2-weighted images; (3) primary tumors with no history of craniotomy, stereotactic biopsy or radiotherapy. Exclusion criteria were: (1) with other neurological disorders (e.g., stroke, trauma) or idiopathic epilepsy that could confound seizure evaluation; (2) unavailable preoperative MR images or poor imaging quality; (3) unevaluable preoperative seizure frequency or types. To maintain the scientific integrity of the present study, we here provide a brief description of the image acquisition and tumor segmentation that was detailed in our previous report (Wang et al., 2015). This study was approved by the institutional ethics committee of local institute, and the requirement for written informed consent was waived due to the study’s retrospective nature.

### 2.2 Evaluation of tumor-related seizures and seizure outcomes

Seizure frequency and types were evaluated by a senior epileptologist according to the updated classification of the International League Against Epilepsy (Beniczky et al., 2025). The seizure frequency was graded as daily (≥1/day), weekly (≥1/week), monthly (≥1/month) or rarely (<1/month) occurrence during the 3 months prior to surgery. The seizures that presented more than once per month were considered a frequently occurring seizure while seizures that presented less than once per month were considered to be non-frequently occurred. The seizure outcome was measured at 6 months after surgery according to the Engel Classification (Engel, 2001).

### 2.3 Brain imaging and tumor segmentation

The majority of the patients were scanned on a 3.0 Tesla MR scanner (Magnetom Trio, Siemens AG, Erlangen, Germany) (*n* = 268). Other MR images were acquired on a Magnetom Verio 3.0 Tesla scanner (Siemens, Erlangen, Germany) or HD 1.5 Tesla scanner (GE Medical System, Waukesha, USA). Brain tumors were manually segmented on each patient’s T2-weighted image using MRIcro by two experienced neurosurgeons who were blinded to the clinical information of the patients. If a larger inconsistency (>5%) existed, a senior neuroradiologist determined the mask to be used in this study. All tumor masks were registered to the standard MNI brain template.

### 2.4 Voxel based neuroimaging analysis

The VLSM analysis was based on applying the General Linear Model (Y = *β*X + *ε*) to each and every individual voxel in the template brain space (Bates et al., 2003). For each voxel, Y represents the lesion involvement (1 = involvement and 0 = tumor free) in every patient, and X represents the symptom matrix. This regression model computed the t-statistic value for *β*, which indicates the correlation of the voxel to the frequency of seizure occurrence. ε is the estimated residual. The scores used in the first column of X were based on the frequency of seizures during the 3 months prior to surgery: 0 - no history of a seizure; 1 - history of only one or two seizures; 2 - more than once per month; 3 - more than once per week; and 4 - more than once per day. The second, third, and fourth columns of X represent the sex, age, and tumor volume of each patient, respectively. The effect of these three factors in the regression model was regressed out from the result. The statistical threshold was determined for each voxel based on a permutation testing (*n* = 1,000). The *t*-values of the voxels that were greater than the *t*-values in >95% of the permutations were preserved in the results (power > 0.8, alpha set at 0.05) (Kimberg et al., 2007). Tumor involvement was defined as a segmented tumor mask that contained the voxel with the highest *t*-value in the VLSM-identified cluster.

### 2.5 Statistical analysis

To identify the clinical characteristics associated with the seizure frequency, univariate analyses were performed using the chi-square test for dichotomous variables and the Mann–Whitney U-test for continuous nonparametric variables. Multivariate logistic regression was performed by entering the significant variables (*p* < 0.05) in the univariate analysis. Engel classification was dichotomized as Class I (seizure free) and Classes II–IV (uncontrolled seizures) in the multivariate analysis.

## 3 Results

### 3.1 Clinical characteristics and tumor distribution

The main clinical and radiological characteristics of the patients are demonstrated (Table 1). The majority of brain regions had large numbers of overlapping tumor masks. Additionally, a statistical power map was computed to identify the brain regions with sufficient statistical power to examine the reliable correlations (see Supplementary Figure S1, which illustrates the computed power map) (Kimberg et al., 2007). Preoperative ASM use in the patients is listed (see Supplementary Table S1, which demonstrates the ASM use).

**Table 1:** Clinical characteristics of the patients (*n* = 352).

Characteristics	Seizures				No seizure	*p*-value^b^
	Total	Frequent	Non-frequent	*p*-value^a^		
Number of patients	246	126	120		106	
Age, Median (range)	37 (16–68)	37 (16–64)	37 (21–68)	0.977	39 (17–61)	0.245
>40 yrs	86	43	43	0.779	48	0.067
Male	154	83	71	0.277	58	0.166
Location						
Hemisphere L/R	145/101	72/54	73/47	0.556	45/61	**0.004**
Frontal	178	89	89	0.536	60	**0.004**
Temporal	69	38	31	0.450	40	0.071
Insula	42	25	17	0.237	18	0.983
Parietal	36	20	16	0.572	13	0.556
Occipital	4	3	1	0.337	2	0.862
Tumor size (Mean ± SD, cm^3^)	80.8 ± 60.0	83.7 ± 62.4	77.7 ± 56.5	0.437	93.0 ± 75.0	0.106
Seizure type						
Focal aware	52	40	12	**<0.001**	NA	
Focal impaired awareness	26	15	11	0.485	NA	
Focal to bilateral tonic–clonic	168	71	97	**<0.001**	NA	
Seizure frequency (in 3 m prior to surgery)						
1–2 seizures	120	0	120		NA	
≥1/month	75	75	0		NA	
≥1/week	25	25	0		NA	
≥1/day	26	26	0		NA	
ASM treatment					NA	
Yes/No	239/7	126/0	113/7	**0.006**	31/75	**<0.001**
Extent of resection						
Gross total	110	46	64	**0.008**	55	0.216
Partial	136	80	56		51	
Tumor pathology						
Oligodendroglioma	45	27	18	0.192	21	0.738
Astrocytoma	85	44	41	0.901	38	0.815
Oligoastrocytoma	116	55	61	0.259	47	0.627

a Comparison between patients with frequent versus non-frequent seizure occurrences.

b Comparison between patients with a history versus no history of seizures.

NA, not available; ASM, anti-seizure medication.

Bold values indicate statistical significance (*p* < 0.05).

### 3.2 VLSM analysis findings

**Figure 1:**
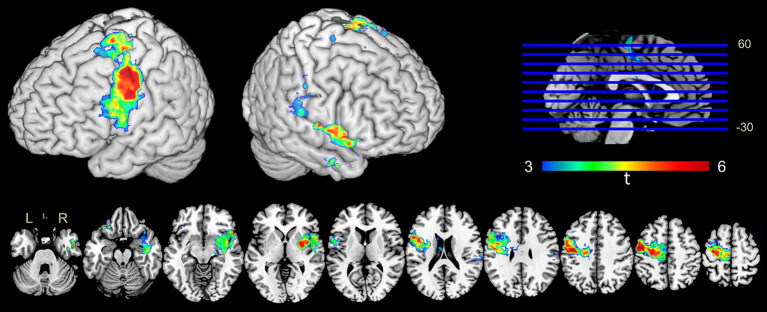
Voxel-based anatomical correlates of seizure frequency following tumor involvement. The color range represents the *t*-values. Voxels with a high correlation effect were predominantly located in the left primary motor area and right insula and the basil ganglia area.

Anatomic correlates of seizure frequency were identified by the VLSM approach (Figure 1). The voxels that were significantly correlated with frequently occurred seizures were preferentially located in the left primary motor area and right insula and the basal ganglia. The voxels with the highest *t*-value in the left (t_max_ = 5.7, x = 115, y = 113, z = 122) and the right (t_max_ = 5.0, x = 65, y = 129, z = 73) hemispheres had the strongest effects.

### 3.3 Predisposing factors to tumor-related seizures

The clinical characteristics and MRI features were compared between the patients who had preoperative seizures and the patients who had no seizures. In the univariate analysis (Table 1), a left-hemispheric tumor (*p* = 0.004) and a frontal lobe location (*p* = 0.004) were associated with the presence of seizures. These variables remained significant in the multivariate analysis, which indicated that the patients with left-hemispheric (*p* = 0.017, OR = 1.774) and frontal located tumors (*p* = 0.015, OR = 1.821) were more likely to present with seizures compared with the other patients.

### 3.4 Factors associated with seizure frequency

**Figure 2:**
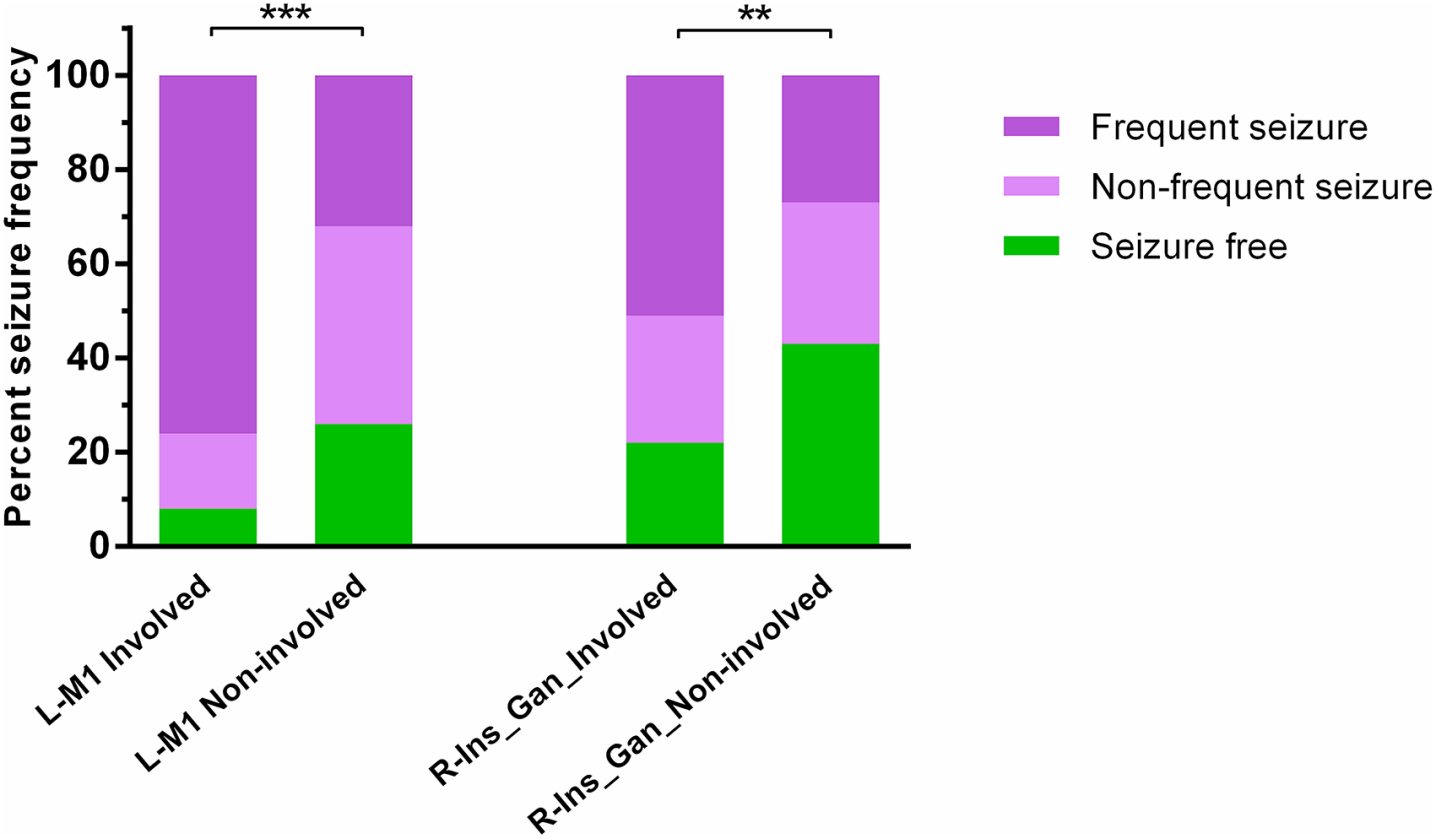
Comparison of the frequency of seizure occurrence in patients with low-grade gliomas that did or did not involve VLSM-localized regions. Tumors involving the left primary motor area (L-M1) were more likely to cause frequent seizures than left-hemispheric tumors located elsewhere (*p* < 0.001, Mann–Whitney *U*-test). A similar significance was identified between the tumors that did or did not involve the right insula and basal ganglia (R-Ins_Gan, *p* = 0.003, Mann–Whitney *U*-test). **p = 0.003; ***p < 0.001.

Clinical characteristics were compared between the patients with frequently (≥ 1/month) and non-frequently (< 1/month) occurred seizures (Table 1). A strong association between the tumor location and seizure frequency was identified (Figure 2). LGGs that involved the VLSM-localized region in the left primary motor area were significantly more likely to cause frequent seizures than left-hemispheric LGGs located in other brain regions (*p* < 0.001). Similar significant differences were identified between the right-hemispheric tumors that did or did not involve the VLSM region in the right insula and the basal ganglia (*p* = 0.003). Furthermore, the seizure type was also associated with the seizure frequency. A focal aware seizure was more likely to occur frequently compared with a focal to bilateral tonic–clonic seizure in patients with LGGs (*p* < 0.001). Of note, the gross-total surgical resection was less likely to be achieved in the patients with LGGs that caused frequently occurred seizures (*p* = 0.008, chi square test).

### 3.5 Factors associated with seizure outcome

Seizure control at 6 months after surgery was available in 185 patients with preoperative seizures. Notably, the frequent occurrence of seizures prior to surgery was strongly associated with a worse seizure outcome in patients with LGGs (*p* = 0.002) (Supplementary Table S2). In addition, a focal aware seizure (*p* < 0.001), oligodendroglioma (*p* = 0.029), and partial resection (*p* < 0.001) were also associated with a worse seizure outcome in the univariate analysis. In the multivariate logistic regression, focal aware seizures (*p* < 0.001, OR = 2.053) and tumor residual (*p* < 0.001, OR = 5.031) predicted uncontrolled seizures at 6 months after surgery.

## 4 Discussion

This is a clinical investigation to explore the anatomic correlation between tumor involvement and seizure frequency in a large cohort of LGG population. Moreover, a voxel-based regression analysis of MR images was used as the basis for the correlation. Notably, we demonstrated that the patients with LGGs that involved in the left primary motor area and the right insula and basal ganglia had an increased risk of the frequent occurrence of epileptic seizures.

A variation in seizure susceptibility has been identified in different brain regions (Westermark et al., 2025). The frontal lobe tumors, especially for those located in the eloquent regions, were found to be associated with a higher risk of seizures (Pallud et al., 2014; McGonigal, 2022; Audrey et al., 2024). The current study demonstrated that the left primary motor area was strongly associated with a high frequency of epileptic seizures. Remarkably, voxels with high statistical significance were preferentially located in the middle and ventral portions of the primary motor area, which are the motor representative areas for the hands and the face. The susceptibility of these regions to seizure was consistent with the clinical observations that epileptic symptoms often present as convulsions of the hand or face. Therefore, this study furthers the current understanding of the role of functional areas in tumor-related seizures that suggests LGGs involved in the dominant motor area are not only associated with an increased risk of seizure, but are also prone to lead to frequent occurrence of seizures. Additionally, the right insula and the basal ganglia were localized as susceptible areas to frequent seizures in the patients with right hemispheric LGGs.

The significant association between tumor location and seizure frequency does not guarantee that the epileptogenic foci are localized in the tumors. On the contrary, tumors can cause seizures by changing the peri-tumoral environment, and the epileptogenic foci that correspond to tumor location were only identified in approximately two-thirds of the patients (Adhikari et al., 2021). Meanwhile, studies applying magnetoencephalography suggested that alterations of functional-connectivity network were a major contributor to tumor-related seizures (Douw et al., 2010; Bonilha et al., 2015; Zimmermann et al., 2024).

In addition to tumor location, several clinical factors have been associated with LGG-related seizures, including age, gender, Karnofsky performance scale, IDH1 mutation, increased intracranial pressure, and cortex involvement (Yuan et al., 2013; Chen et al., 2017; Li et al., 2018; Dantio et al., 2025). Nevertheless, no clear relationship has been established between tumor characteristics and seizure frequency (Berntsson et al., 2018). The current study shows the seizure type was associated with the seizure frequency that focal aware seizures were more likely to occur frequently than focal to bilateral tonic–clonic seizures (*p* < 0.001). Remarkably, LGGs that caused frequently occurred seizures were less likely to be completely removed, which may be attributed to the higher incidence of the involvement of these tumors in functional brain regions, such as the primary motor area (Figure 2).

The factors associated with seizure control in patients with LGG remain incompletely understood. A shorter history of seizure appears to be associated with a favorable prognosis for seizure control (Le et al., 2023). In contrast, a longer seizure history and focal aware seizures in patients with LGGs have predicted poor seizure control (Neal et al., 2016; Shan et al., 2018). Gross-total resection of LGG was identified as a critical predictor for complete seizure freedom following surgery (Still et al., 2018; Ius et al., 2020; He et al., 2023). Both radiotherapy and chemotherapy were identified to be associated with the improvement of seizure control in patients with LGG (Koekkoek et al., 2015; Shan et al., 2018). In this study, univariate analysis identified the significant predictors for worse seizure outcome, which included a focal aware seizure, a high seizure frequency prior to surgery, oligodendrogliomas, and an incomplete surgical resection. The seizure type and extension of resection continued to have a strong impact on seizure outcome in the multivariate analysis. Notably, the current study demonstrated that LGG patients with frequent seizures were less likely to achieve gross-total resection, which may explain the worse seizure outcome of these patients.

There were several limitations to the current study. First, 24 h video-electroencephalography monitoring was not performed in this study because only a very small proportion of the LGG patients were expected to experience a daily occurrence of seizures; thus, mild-symptomatic seizures with epileptic discharges may have been missed. Variations between individual cases may have existed in the preoperative seizure control as a result of the use of ASMs, which could not be teased apart in the current study. Nevertheless, despite these limitations, the results reported here were based on statistical quantitative analysis in a large number of patients that achieved an objective neuroimaging interpretation. In the future, researchers are encouraged to utilize a prospective design that includes the use of repeated video-electroencephalography examinations prior to surgical treatment, so that neurophysiological evidence of the presence of epileptic discharge can be acquired.

## 5 Conclusion

In conclusion, we identified two specific brain regions that correlated with a frequent occurrence of tumor-related seizures. Patients with LGGs that involved the left primary motor area or right insula and the basal ganglia were more likely to experience a high frequency of seizures.

## Data Availability

The original contributions presented in the study are included in the article/Supplementary material, further inquiries can be directed to the corresponding authors.

## Footnotes

1
http://www.cgga.org.cn

## References

[ref1] Adhikari S. Walker B. C. Mittal S. (2021). “Pathogenesis and management of brain tumor-related epilepsy,” in Gliomas, ed. Debinski W. (Brisbane (AU): Exon Publications). 34038047

[ref2] Akeret K. Stumpo V. Staartjes V. E. Vasella F. Velz J. Marinoni F. . (2020). Topographic brain tumor anatomy drives seizure risk and enables machine learning based prediction. Neuroimage Clin. 28:102506. doi: 10.1016/j.nicl.2020.102506, 33395995 PMC7711280

[ref3] Audrey C. Lim K.-S. Ahmad Zaki R. Narayanan V. Fong S.-L. Tan C.-T. (2024). From location to manifestation: a systematic review and meta-analysis of seizure prevalence in different brain tumor sites. Brain Disord. 14:100146. doi: 10.1016/j.dscb.2024.100146

[ref4] Bates E. Wilson S. M. Saygin A. P. Dick F. Sereno M. I. Knight R. T. . (2003). Voxel-based lesion-symptom mapping. Nat. Neurosci. 6, 448–450. doi: 10.1038/nn1050, 12704393

[ref5] Beniczky S. Trinka E. Wirrell E. Abdulla F. Al Baradie R. Alonso Vanegas M. . (2025). Updated classification of epileptic seizures: position paper of the international league against epilepsy. Epilepsia 66, 1804–1823. doi: 10.1111/epi.18338, 40264351 PMC12169392

[ref6] Berntsson S. G. Merrell R. T. Amirian E. S. Armstrong G. N. Lachance D. Smits A. . (2018). Glioma-related seizures in relation to histopathological subtypes: a report from the glioma international case-control study. J. Neurol. 265, 1432–1442. doi: 10.1007/s00415-018-8857-0 29687214 PMC5990563

[ref7] Bonilha L. Jensen J. H. Baker N. Breedlove J. Nesland T. Lin J. J. . (2015). The brain connectome as a personalized biomarker of seizure outcomes after temporal lobectomy. Neurology 84, 1846–1853. doi: 10.1212/WNL.0000000000001548 25854868 PMC4433467

[ref8] Chen H. Judkins J. Thomas C. Wu M. Khoury L. Benjamin C. G. . (2017). Mutant IDH1 and seizures in patients with glioma. Neurology 88, 1805–1813. doi: 10.1212/WNL.0000000000003911 28404805 PMC5419985

[ref9] Dantio C. D. Fasoranti D. O. Teng C. Li X. (2025). Seizures in brain tumors: pathogenesis, risk factors and management (review). Int. J. Mol. Med. 55, 1–18. doi: 10.3892/ijmm.2025.5523, 40116082 PMC11964414

[ref10] Douw L. Van Dellen E. De Groot M. Heimans J. J. Klein M. Stam C. J. . (2010). Epilepsy is related to theta band brain connectivity and network topology in brain tumor patients. BMC Neurosci. 11:103. doi: 10.1186/1471-2202-11-103, 20731854 PMC2936439

[ref11] Engel J. Jr. (2001). A proposed diagnostic scheme for people with epileptic seizures and with epilepsy: report of the ILAE task force on classification and terminology. Epilepsia 42, 796–803. doi: 10.1046/j.1528-1157.2001.10401.x 11422340

[ref12] Feyissa A. M. Sanchez-Boluarte S. S. Moniz-Garcia D. Chaichana K. L. Sherman W. J. Freund B. E. . (2023). Risk factors for preoperative and postoperative seizures in patients with glioblastoma according to the 2021 World Health Organization classification. Seizure 112, 26–31. doi: 10.1016/j.seizure.2023.09.013, 37729723

[ref13] Grimi A. Bono B. C. Lazzarin S. M. Marcheselli S. Pessina F. Riva M. (2024). Gliomagenesis, epileptogenesis, and remodeling of neural circuits: relevance for novel treatment strategies in low- and high-grade gliomas. Int. J. Mol. Sci. 25:8953. doi: 10.3390/ijms25168953, 39201639 PMC11354416

[ref14] Hardstone R. Ostrowski L. M. Dusang A. N. Lopez-Larraz E. Jesser J. Cash S. S. . (2025). Extension of voxel-based lesion mapping to multidimensional neurophysiological data. Sci. Rep. 15:41488. doi: 10.1038/s41598-025-17247-z, 41274911 PMC12644473

[ref15] He X. Zhang K. Liu D. Yang Z. Li X. Yang Z. (2023). Predictors of seizure outcomes in patients with diffuse low-grade glioma-related epilepsy after complete glioma removal. CNS Neurosci. Ther. 29, 736–743. doi: 10.1111/cns.14061, 36514187 PMC9873512

[ref16] Ius T. Pauletto G. Tomasino B. Maieron M. Budai R. Isola M. . (2020). Predictors of postoperative seizure outcome in low grade glioma: from volumetric analysis to molecular stratification. Cancers (Basel) 12:397. doi: 10.3390/cancers12020397, 32046310 PMC7072647

[ref17] Jiang T. Nam D. H. Ram Z. Poo W. S. Wang J. Boldbaatar D. . (2026). Updated clinical practice guidelines for the management of adult diffuse gliomas. Cancer Lett. 640:218185. doi: 10.1016/j.canlet.2025.218185, 41338443

[ref18] Kimberg D. Y. Coslett H. B. Schwartz M. F. (2007). Power in voxel-based lesion-symptom mapping. J. Cogn. Neurosci. 19, 1067–1080. doi: 10.1162/jocn.2007.19.7.1067, 17583984

[ref19] Koekkoek J. A. Kerkhof M. Dirven L. Heimans J. J. Reijneveld J. C. Taphoorn M. J. (2015). Seizure outcome after radiotherapy and chemotherapy in low-grade glioma patients: a systematic review. Neuro-Oncology 17, 924–934. doi: 10.1093/neuonc/nov032, 25813469 PMC5654353

[ref20] Le V. T. Nguyen A. M. Pham T. A. Nguyen P. L. (2023). Tumor-related epilepsy and post-surgical outcomes: tertiary hospital experience in Vietnam. Sci. Rep. 13:10859. doi: 10.1038/s41598-023-38049-1, 37407622 PMC10322847

[ref21] Li J. Long S. Zhang Y. Wei W. Yu S. Liu Q. . (2024). Molecular mechanisms and diagnostic model of glioma-related epilepsy. NPJ Precis. Oncol. 8:223. doi: 10.1038/s41698-024-00721-8, 39363097 PMC11450052

[ref22] Li Y. Shan X. Wu Z. Wang Y. Ling M. Fan X. (2018). IDH1 mutation is associated with a higher preoperative seizure incidence in low-grade glioma: a systematic review and meta-analysis. Seizure 55, 76–82. doi: 10.1016/j.seizure.2018.01.011 29414139

[ref23] Mazzucchi E. Vollono C. Pauletto G. Lettieri C. Budai R. Gigli G. L. . (2022). The persistence of seizures after tumor resection negatively affects survival in low-grade glioma patients: a clinical retrospective study. J. Neurol. 269, 2627–2633. doi: 10.1007/s00415-021-10845-7, 34693462

[ref24] McGonigal A. (2022). Frontal lobe seizures: overview and update. J. Neurol. 269, 3363–3371. doi: 10.1007/s00415-021-10949-0, 35006387

[ref25] Neal A. Morokoff A. O'brien T. J. Kwan P. (2016). Postoperative seizure control in patients with tumor-associated epilepsy. Epilepsia 57, 1779–1788. doi: 10.1111/epi.13562, 27666131

[ref26] Pallud J. Audureau E. Blonski M. Sanai N. Bauchet L. Fontaine D. . (2014). Epileptic seizures in diffuse low-grade gliomas in adults. Brain 137, 449–462. doi: 10.1093/brain/awt345 24374407

[ref27] Ryden I. Thurin E. Carstam L. Smits A. Gulati S. Henriksson R. . (2021). Psychotropic and anti-epileptic drug use, before and after surgery, among patients with low-grade glioma: a nationwide matched cohort study. BMC Cancer 21:248. doi: 10.1186/s12885-021-07939-w, 33685410 PMC7938599

[ref28] Shan X. Fan X. Liu X. Zhao Z. Wang Y. Jiang T. (2018). Clinical characteristics associated with postoperative seizure control in adult low-grade gliomas: a systematic review and meta-analysis. Neuro-Oncology 20, 324–331. doi: 10.1093/neuonc/nox130, 29016869 PMC5817956

[ref29] Slegers R. J. Blumcke I. (2020). Low-grade developmental and epilepsy associated brain tumors: a critical update 2020. Acta Neuropathol. Commun. 8:27. doi: 10.1186/s40478-020-00904-x, 32151273 PMC7063704

[ref30] Still M. E. H. Roux A. Huberfeld G. Bauchet L. Baron M. H. Fontaine D. . (2018). Extent of resection and residual tumor thresholds for postoperative total seizure freedom in epileptic adult patients harboring a supratentorial diffuse low-grade glioma. Neurosurgery 85, E332–E340. doi: 10.1093/neuros/nyy481, 30395304

[ref31] Wang Y. Qian T. You G. Peng X. Chen C. You Y. . (2015). Localizing seizure-susceptible brain regions associated with low-grade gliomas using voxel-based lesion-symptom mapping. Neuro-Oncology 17, 282–288. doi: 10.1093/neuonc/nou130, 25031032 PMC4288515

[ref32] Wang Y. Wang Y. Bao H. Wang Z. Jiang T. (2025). Mechanisms and therapeutic implications of glioma-neuron interactions. Cancer Lett. 629:217884. doi: 10.1016/j.canlet.2025.217884, 40545026

[ref33] Westermark A. Fahlstrom M. Mirza S. Zetterling M. Kumlien E. Latini F. (2025). Subcortical brain regions associated with seizure risk in patients with idh mutated diffuse gliomas. Brain Behav. 15:e70477. doi: 10.1002/brb3.70477, 40200848 PMC11979491

[ref34] You G. Sha Z. Jiang T. (2020). Clinical diagnosis and perioperative management of glioma-related epilepsy. Front. Oncol. 10:550353. doi: 10.3389/fonc.2020.550353, 33520690 PMC7841407

[ref35] Yuan Y. Xiang W. Yanhui L. Ruofei L. Shuang L. Yingjun F. . (2013). Ki-67 overexpression in WHO grade II gliomas is associated with poor postoperative seizure control. Seizure 22, 877–881. doi: 10.1016/j.seizure.2013.08.004, 23992787

[ref36] Zimmermann M. L. M. Breedt L. C. Centeno E. G. Z. Reijneveld J. C. Santos F. A. N. Stam C. J. . (2024). The relationship between pathological brain activity and functional network connectivity in glioma patients. J. Neuro-Oncol. 166, 523–533. doi: 10.1007/s11060-024-04577-7, 38308803 PMC10876827

