# Supplementary material for: Brain regions associated with frequent occurrences of seizures related to low-grade gliomas: a voxel-level evidence
Source: Front Hum Neurosci. 2026 Mar 27;20:1792225. doi: 10.3389/fnhum.2026.1792225 (PMC13066257; doi:10.3389/fnhum.2026.1792225)
Supplement: Supplementary file 1 [file Data_Sheet_1.docx]

Supplementary Material

# Supplementary Figures and Tables

##
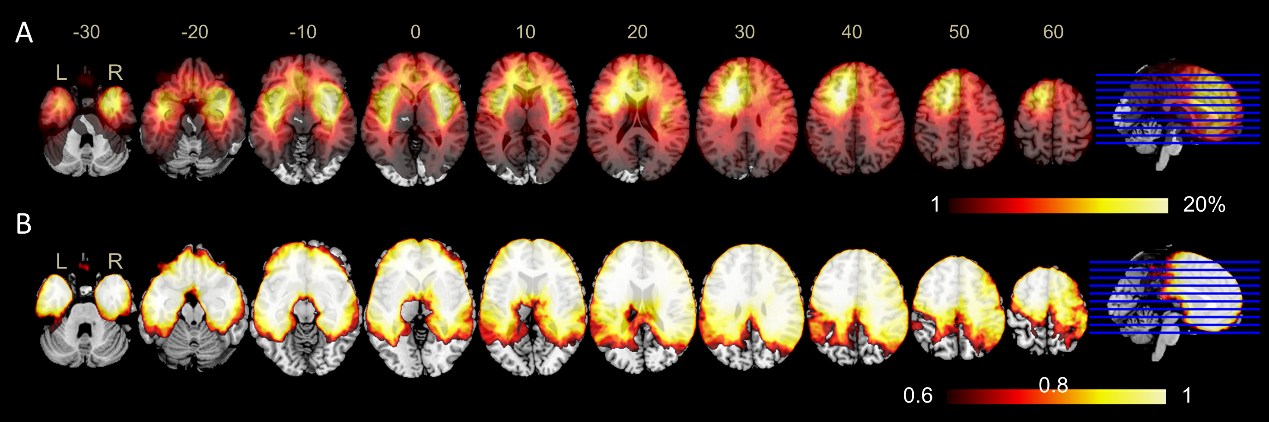
Supplementary Figures

**Supplementary Figure 1.** (A) Overlap of all tumor lesions (n = 352). Tumor overlaps are indicated by color ranging from low (dark, one case overlaps) to high (bright, more than 20 % of cases overlap). (B) Power map for voxel-based lesion symptom analysis where the color map shows the distribution of power, ranging from 0.6 (dark red) to 1 (light yellow), with alpha set to p < 0.05. Only voxels with high power values (> 0.8) were included in the voxel-based lesion symptom analysis. MNI space brain template was used to display the result.

## Supplementary Tables

**Supplementary Table S1**. **Preoperative ASM use in the patients (*n* = 352)**

| **Treatment** | **Seizures** | | | **Seizure free** |
| --- | --- | --- | --- | --- |
|  | total | frequent | non-frequent |  |
| Number of patients | 246 | 126 | 120 | 106 |
| Any ASMs | 239 | 126 | 113 | 31 |
| Valproic acid | 207 | 108 | 99 | 29 |
| Carbamazepine | 3 | 1 | 2 | 1 |
| Phenytoin | 2 | 2 | 0 | 0 |
| Combination | 27 | 15 | 12 | 1 |

**Supplementary Table S2. Clinical predictors of seizure control (6 months after surgery).**

| **Characteristics** |  | **Univariate^a^** | |  |  | **Multivariate^b^** |  |
| --- | --- | --- | --- | --- | --- | --- | --- |
|  | **Total** | **Engel I** | **Engel II-IV** | **p-value** |  | **OR(95% CI)** | **p-value** |
| **Number of patients** | 185 | 123 | 62 |  |  |  |  |
| **Age** (>40 yrs) | 62 | 39 | 23 | 0.464 |  |  |  |
| **Sex(M/F)** | 113/72 | 74/49 | 39/23 | 0.752 |  |  |  |
| **Location** |  |  |  |  |  |  |  |
| Hemisphere L/R | 126/59 | 84/39 | 42/20 | 0.940 |  |  |  |
| VLSM-region involved |  |  |  |  |  |  |  |
| Yes/No | 43/142 | 28/95 | 15/47 | 0.828 |  |  |  |
| Frontal involvement |  |  |  |  |  |  |  |
| Yes/No | 137/48 | 93/30 | 44/18 | 0.497 |  |  |  |
| **Seizure type** |  |  |  |  |  |  |  |
| Focal aware | 50 | 21 | 29 | **<0.001** |  | 2.053(1.395-3.021) | **<0.001** |
| Focal impaired awareness | 23 | 15 | 8 |  |  |  |  |
| Focal to bilateral tonic-clonic | 112 | 87 | 25 |  |  |  |  |
| **Seizure frequency**  (in 3m prior to surgery) |  |  |  |  |  |  |  |
| ≥1/month | 96 | 54 | 42 | **0.002** |  | 1.539(0.742-3.195) | 0.247 |
| <1/month | 89 | 69 | 20 |  |  |  |  |
| **ASM Treatment** |  |  |  |  |  |  |  |
| Yes/No | 185/0 | 123/0 | 62/0 |  |  |  |  |
| **Extent of resection** |  |  |  |  |  |  |  |
| Partial | 107 | 56 | 51 | **<0.001** |  | 5.031(2.334-10.846) | **<0.001** |
| Gross total | 78 | 67 | 11 |  |  |  |  |
| **Tumor pathology** |  |  |  |  |  |  |  |
| Oligodendroglioma | 27 | 13 | 14 | **0.029** |  | 2.322(0.905-5.956) | 0.080 |
| Astrocytoma | 76 | 54 | 22 | 0.272 |  |  |  |
| Oligoastrocytoma | 82 | 56 | 26 | 0.642 |  |  |  |

^a^ Results of chi-square test.

^b^ Results of logistic regression analysis.

CI, confidence interval; OR, odds ratio; ASM, anti-seizure medication.
